# Medicine Men of the World.—II

**Published:** 1889-05-04

**Authors:** 


### MEDICINE MEN OF THE WORLD.—II.

THE sufferings of childhood are probably a good deal less among savages than they are in many civilised communities, and that for at least one good substantial reason. With an ailing or injured child. over a great part of the earth, it is a case of kill or cure. Not that savages are half so prone to infanticide as has very often been made out. Many of the accounts given of the extent of this in various uncivilised countries have undoubtedly been a good deal exaggerated. Still, it is not to be denied that it is by no means an unusual thing for a little barbarian who comes into the world either malformed or sickly to have its life snuffed out by deliberate purpose. The mothers, poor things—there is a deal of human nature in them whatever skins they may wear—may resent it or resist it with frantic vehemence, but if it does not appear to be for the good of the tribe that a child shall be allowed to live, it is a very common thing for it to die, and thus it happens that barbarian babies are not often found afflicted as many among ourselves are.

But though they be not killed of set purpose, babies who contract any kind of disorder of a serious character had need have a good sound constitution to stand the doctoring they are pretty sure to come in for at the hands of the medicine men. Even a perfectly healthy child, over which the parents may be disposed to rejoice ever so greatly, has in some parts of the world a good deal to put up with. Among the Kaffirs, for instance, unless things have been a good deal changed of late years—and in writing or thinking of barbarians it is always worth while to remember that in these days of general intercommunication many of them have, within the past generation or two, very greatly changed where they have not died out altogether; but among the Kaffirs the medicine man, if he is at all what he used to be, is a terrible torment to babydom. When a Kaffir woman becomes a mother the doctor pays her no attention whatever. He is supposed to be called in for the child only, and he accordingly devotes his whole attention to the little darkey—though, by the way, the Kaffir is not born a darkey, but almost as white as an ordinary European baby, and acquires the red-black of his parents only in course of time. The medicine man is a mighty deliberate personage among the Kaffirs, but everything is at a standstill till he comes—the young stranger not being allowed so much as a drop of the lightest of refreshment till he has been seen by the great man. When the doctor comes, the first thing he does is to make a number of incisions in various parts of the child's body, very much as he would do if he were going to vaccinate it. Then he rubs in "medicine," though the unfortunate little urchin may be in perfect health. This is allowed to operate—goodness only knows how—till the next day, when the doctor calls again, deepens the incisions a little, and rubs in more medicine. Then at length the child is washed, and dried by being moved to and fro in the smoke of a wood fire; and if it survive—which many a new-born infant might find some difficulty in doing, for a Kaffir baby is often reduced to the verge of starvation before the services of the doctor can be secured—it is daubed all over with red paint, which, during the early months of his troubled career, is renewed as often as a smart appearance may seem to require. The Kaffirs are reckoned among the most intelligent of African races; but their doctors are terrible fellows, and the degree of sagacity they bring to bear on all medical matters may be gathered from their procedure in the case of new-born infants.

The young Kaffir has at least an advantage over some other tiny barbarians. He is permitted the free use of his limbs. Even in sickness many of the North American Indian babies are bound firmly down to a sort of backboard. This is the case with the Sioux, for instance. A flat board is padded all over with dry moss or soft grass, and the body of the child is bound down upon it with cords of plaited grass or thongs of leather, while flaps of dressed skin are laced over all but the face of the infant, and it is then disposed of for 20 or four-and-twenty hours. If it should fall sick it is not released from its rigorous bondage and fondled and nursed as are our pampered little aristocrats, though the Indian mother gives many touching indications of her tender love for her child. She anxiously does the best she can for her little one, but here, as in so many other dark corners of the earth, sickness is not a mere physical derangement. It is the doing of an evil spirit, and if all that the mother can think of proves insufficient to dislodge the demon that has taken possession of the little sufferer, there is nothing for it but to call in the "ghaman," or female doctor. Into her merciless hands the backboard and the little mortal stripped upon it are given over to have the devil driven out. The usual way of exorcising the evil spirit is by dragging and dancing the little sufferer about, and making all the most hideous and appalling noises that yelling lungs and calabashes and rattles can yield by day and night without intermission. It happens, of course, sometimes that a little mortal of vigorous constitution will pull through its troubles in spite of the ghaman's doctoring, which, besides the perpetual din and rough usage, sometimes includes a severe fumigation with the smoke of certain medicinal sticks and herbs. Should the child recover its wonted health under the orthodox treatment, it is, of course, a great triumph for the ghaman, who receives unbounded gratitude and liberal pay. On the other hand, if the child dies, it proves conclusively the malignant character of the evil spirit, and the female doctor gets everywhere great credit for her desperate struggle with a devil of such potency.

Very touching are some of the doings of the mothers of these barbarian bairns when, after all that the medicine men and witch doctors can do, they fly away into the land of spirits. Among the Chinooks their cradles are like little canoes, and when the child dies the tiny corpse is set afloat in it on some lonely sacred pool, with a little paddle and a ladle to bale it out, and with a store of provisions to last them in their long, long journey to the hunting grounds, in which they are ever after to be happy. In some of the other tribes they bury the little body, but the mother will fill its ornamental cradle with black feathers, and will carry it with her wherever she goes, however long her journeys may be, or however toilsome the way. For a year or more she will never part with it. So tender is the affection of these poor mothers for their children, and so vivid is the imagination of these grown-up infants, that they will stand the cradle against the wall, or hang it on a tree and talk to it, and answer the prattle of the little soul that used to be in it. Aye, aye; they are very human all the world over these mothers, and half, and more than half, the world's barbarism is, after all, little more than our own ignorance and our failure to apprehend what is unfamiliar to us. A short and a sure road to the hearts of all people is open to the medical profession—men or women who will take with them real skill and intelligent sympathy, and will go forth into the wailing and the darkness with light, and health, and healing; and if it be but done in the name and in the spirit of the Great Father of us all, there is a mission which all men can honour and a message which all men can accept.

